# Effects of *Aficamten* on cardiac contractility in a feline translational model of hypertrophic cardiomyopathy

**DOI:** 10.1038/s41598-022-26630-z

**Published:** 2023-01-02

**Authors:** Ashley N. Sharpe, Maureen S. Oldach, Victor N. Rivas, Joanna L. Kaplan, Ashley L. Walker, Samantha L. Kovacs, Darren T. Hwee, Peadar Cremin, Bradley P. Morgan, Fady I. Malik, Samantha P. Harris, Joshua A. Stern

**Affiliations:** 1 Department of Medicine and Epidemiology, School of Veterinary Medicine, University of California-Davis, 2108 Tupper Hall, One Shields Ave, Davis, CA 95616 USA; 2 Research and Non-Clinical Development, Cytokinetics, Inc., South San Francisco, CA USA; 3 Department of Cellular and Molecular Medicine, University of Arizona, Tucson, AZ USA

**Keywords:** Cardiology, Pharmacology, Experimental models of disease, Preclinical research, Translational research

## Abstract

Hypertrophic cardiomyopathy (HCM) is the most prevalent inherited cardiac disease in humans and cats and lacks efficacious pharmacologic interventions in the preclinical phase of disease. LV outflow tract obstruction (LVOTO) is commonly observed in HCM-affected patients and is a primary driver of heart failure symptoms and reduced quality of life. Novel small-molecule cardiac myosin inhibitors target actin-myosin interactions to alleviate overactive protein interactions. A prospective, randomized, controlled cross-over study was performed to evaluate pharmacodynamic effects of two doses (0.3 and 1 mg/kg) of a next-in-class cardiac myosin inhibitor, *aficamten* (CK-3773274, CK-274), on cardiac function in cats with the A31P *MYBPC3* mutation and oHCM. Dose-dependent reductions in LV systolic function, LVOT pressure gradient, and isovolumetric relaxation times compared to baseline were observed. Promising beneficial effects of reduced systolic function warrant further studies of this next-in-class therapeutic to evaluate the benefit of long-term administration in this patient population.

## Introduction

Small molecule inhibitors are an emerging class of novel therapeutic agents that inhibit the function of specific proteins with the potential for neutralizing deleterious downstream effects^1^. One class of these compounds include cardiac myosin inhibitors, which are being investigated for their potential therapeutic uses in a vast array of disease processes including hypertrophic cardiomyopathy (HCM)^2–4^. Hypertrophic cardiomyopathy is the most common heart disease in multiple species and occurs in 0.2% of humans and approximately 14.7% of subclinical domestic cats, with potential complications including congestive heart failure (CHF), arterial thromboembolism (ATE), development of arrythmias and sudden cardiac death (SCD)^2^^,^^5–9^. While many humans and cats may remain asymptomatic for their lifespan, the potential clinical sequelae of disease progression are detrimental and therapies to improve survival are lacking.

HCM is a disease of cardiac sarcomeric proteins with identified mutations in cats affecting the *myosin heavy chain 7* (*MYH7*) and *myosin binding protein-C* (*MYBPC3*) genes, which alter development of the motor protein b-myosin heavy chain and the structural and regulatory protein cardiac myosin binding protein-C, respectively^10^. Sarcomeric proteins are responsible for muscle contraction and relaxation where mutations in these proteins lead to histopathologic changes in the myocardium including myocardial fiber disarray, interstitial fibrosis, and intramural coronary arteriosclerosis^10–12^. These changes result in left ventricular (LV) wall thickening in the absence of another pathogenic explanation (i.e., hyperthyroidism, systemic hypertension, fixed stenosis, metabolic disease, and neoplasia),and predominate hemodynamic alterations include hypercontractility of the LV and diastolic dysfunction which further exacerbate disease progression.

One contributing factor to morbidity and mortality of HCM in human patients is the presence of left ventricular outflow tract obstruction (LVOTO)^13, 14^. The characteristic finding of LVOTO may be secondary to muscular hypertrophy of the basilar interventricular septum or systolic anterior motion of the mitral valve, which is attributed to Venturi forces pulling the septal leaflet into the left ventricular outflow tract (LVOT) or anatomic abnormalities of the mitral valve leaflet itself^15^. Hypertrophic cardiomyopathy with LVOTO is also known as obstructive hypertrophic cardiomyopathy (oHCM), LVOTO is present at rest in approximately 30% of human patients and an additional 30% with exertion and has been documented in up to 64.9% of cats^15, 16^. Hyperdynamic systolic function and LVOTO increase myocardial oxygen consumption that contributes to myocardial ischemia and fibrosis, likely potentiating the development of arrhythmias^17^. Pharmacological or surgical relief of LVOTO is a major target of therapy and significantly improves clinical signs^5^. While LVOTO has not been shown to negatively impact prognosis in cats, its prevalence remains important in the utilization of the cat as a translational model for disease^16^^,^^18^.

Evidence is currently lacking for therapies with proven efficacy in delaying progression of disease or conferring survival benefits in subclinical human and feline HCM patients^19^. The lack of therapeutic options for delaying or preventing harmful outcomes in the preclinical period of disease has prompted investigation into novel therapies targeted at the underlying molecular pathophysiology. A recent study demonstrated the efficacy of another novel small molecule inhibitor in relieving LVOTO in a feline model of oHCM^20^. This small molecule inhibitor also demonstrated efficacy in suppressing histopathologic and gross pathologic changes in a mouse model of HCM, substantiating the promise of these novel therapeutic agents^21^.

The aim of this study was to characterize the pharmacodynamic effects of a single oral administration of two dose strengths (0.3 mg/kg and 1 mg/kg) of the next-in-class cardiac myosin inhibitor, *aficamten*, on cardiac function in purpose-bred cats harboring the naturally occurring A31P *MYBPC3* mutation with a clinical diagnosis of HCM with LVOTO.

## Results

Eight purpose-bred mixed-breed Maine Coon cats (1 intact female/7 intact males) with documented A31P *MYBPC3* mutations (6 heterozygous/2 homozygous positive) and a clinical diagnosis of occult oHCM were enrolled in the study. The mean age of cats was 4.3 $\pm$ 3.0 years. The mean IVS thickness was 6.48 $\pm$ 0.44 and the median LVPW thickness was 5.45 mm (5.10–6.44 mm); patient data is listed in Table 1. All eight cats received vehicle and *aficamten* at a dose of 0.3 mg/kg and 1 mg/kg without any adverse events.

**Table 1:** Cat demographics and maximal left ventricular diastolic wall thickness.

Subject	Sex	Date of Birth	A31P *MYBPC3* Genotype	Max IVSDd (mm)	Max LVPWd (mm)	Date of HCM Confirmation Examination
1	Male	3/1/15	Heterozygous	6.6	5.3	2/3/20
2	Male	2/15/11	Heterozygous	6.2	5.6	2/3/20
3	Female	3/2/15	Homozygous	6.8	5.3	2/3/20
4	Male	1/27/12	Heterozygous	6.2	7.6	2/3/20
5	Male	8/8/17	Heterozygous	7	5.3	2/10/20
6	Male	6/4/19	Homozygous	5.8	6.2	2/10/20
7	Male	4/3/18	Heterozygous	6.2	5.6	2/10/20
8	Male	8/8/17	Heterozygous	7	5.3	2/10/20

*IVSDd* intraventricular septum diameter (diastole), *LVPWd* left ventricular posterior wall (diastole), *HCM* hypertrophic cardiomyopathy.

**Figure 1:**
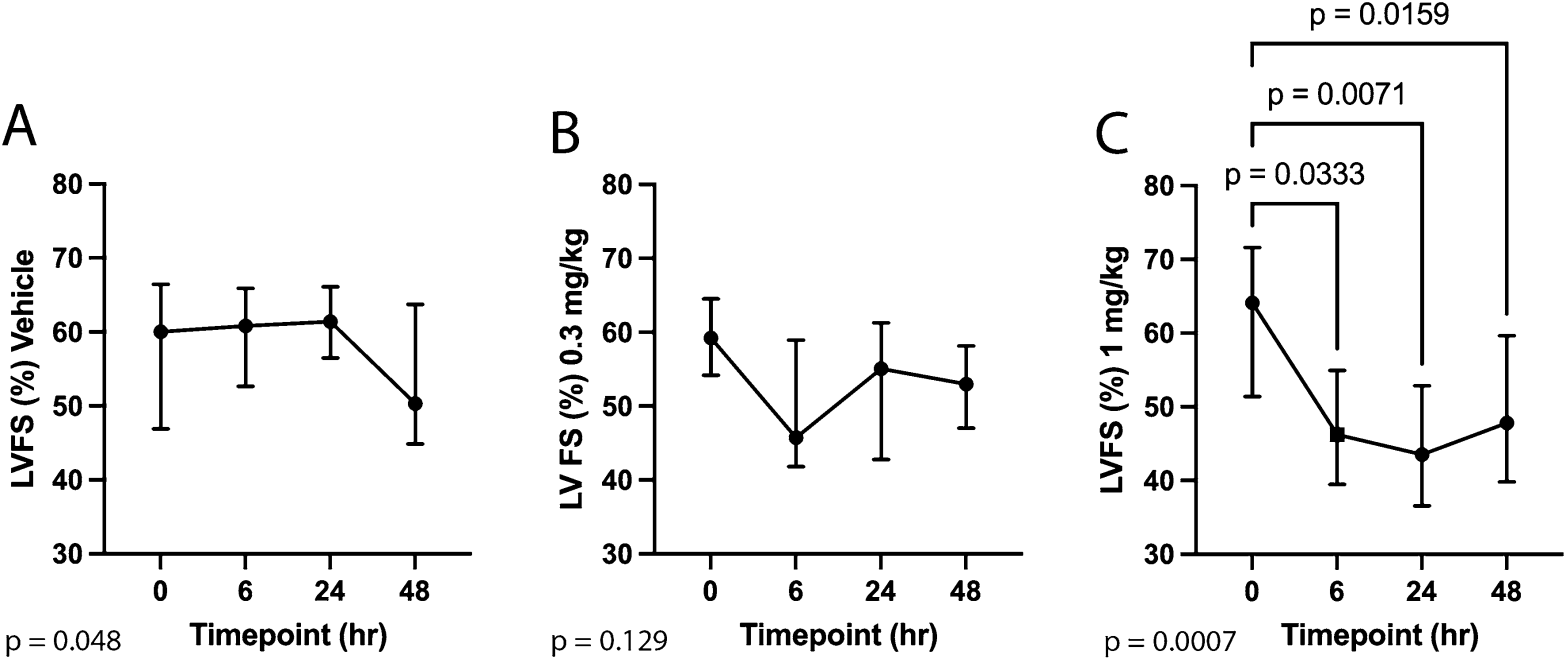
Left ventricular fractional shortening (LV FS) at 6-, 24-, and 48-h post administration of treatment. (**A**) Vehicle. (**B**) *Aficamten* (0.3 mg/kg). (**C**) *Aficamten* (1.0 mg/kg). The median is denoted at each time point and the whiskers represent the interquartile range The overall P value is denoted on the bottom left of each graph. Where pairwise comparisons were significant they are shown with their respective *P* values.

**Figure 2:**
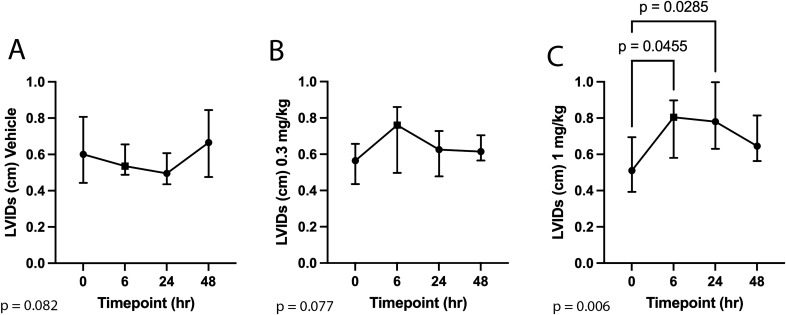
Left ventricular systolic internal dimension (LVIDs) at 6-, 24-, and 48-h post administration of treatment. (**A**) Vehicle. (**B**) *Aficamten* (0.3 mg/kg). (**C**) *Aficamten* (1.0 mg/kg). The median is denoted at each time point and the whiskers represent the interquartile range. The overall P value is denoted on the bottom left of each graph. Where pairwise comparisons were significant they are shown with their respective *P* values.

**Figure 3:**
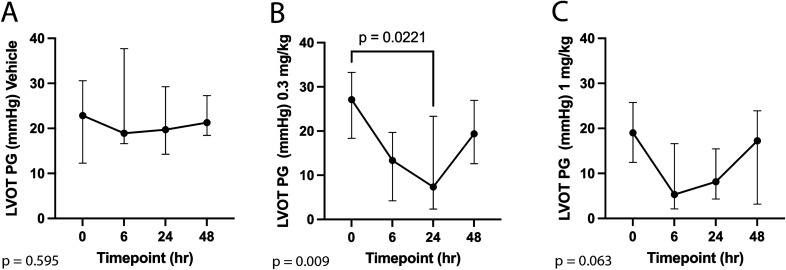
Left ventricular outflow tract peak pressure gradient (LVOT PG) at 6-, 24-, and 48-h post administration of treatment. (**A**) Vehicle. (**B**) *Aficamten* (0.3 mg/kg). (**C**) *Aficamten* (1.0 mg/kg). The median is denoted at each time point and the whiskers represent the interquartile range. The overall P value is denoted on the bottom left of each graph. Where pairwise comparisons were significant they are shown with their respective brackets and *P* values.

Echocardiographic results for the vehicle, 0.3 mg/kg, and 1 mg/kg study groups are reported in Tables 2^,^3, and 4. Administration of *aficamten* at a dose of 1 mg/kg reduced LV FS% from baseline at 6-, 24-, and 48-h post-treatment (*P* = 0.03, *P* = 0.01, *P* = 0.02, respectively; Fig. 1). The reduction in systolic function was due to a decrease in LVIDs at 6- and 24-h post-treatment (*P* = 0.046 and *P* = 0.03, respectively; Fig. 2) without affecting LVIDd (overall *P* = 0.352). *Aficamten* reduced both LVOT peak pressure gradient and incidence of LVOTO at the 0.3 mg/kg. The LVOT peak pressure gradient was reduced with *aficamten* (0.3 mg/kg) treatment overall (*P* = 0.01; Fig. 3) and the incidence of LVOTO was reduced with *aficamten* (1 mg/kg) treatment overall (*P* = 0.01).

**Table 2:**
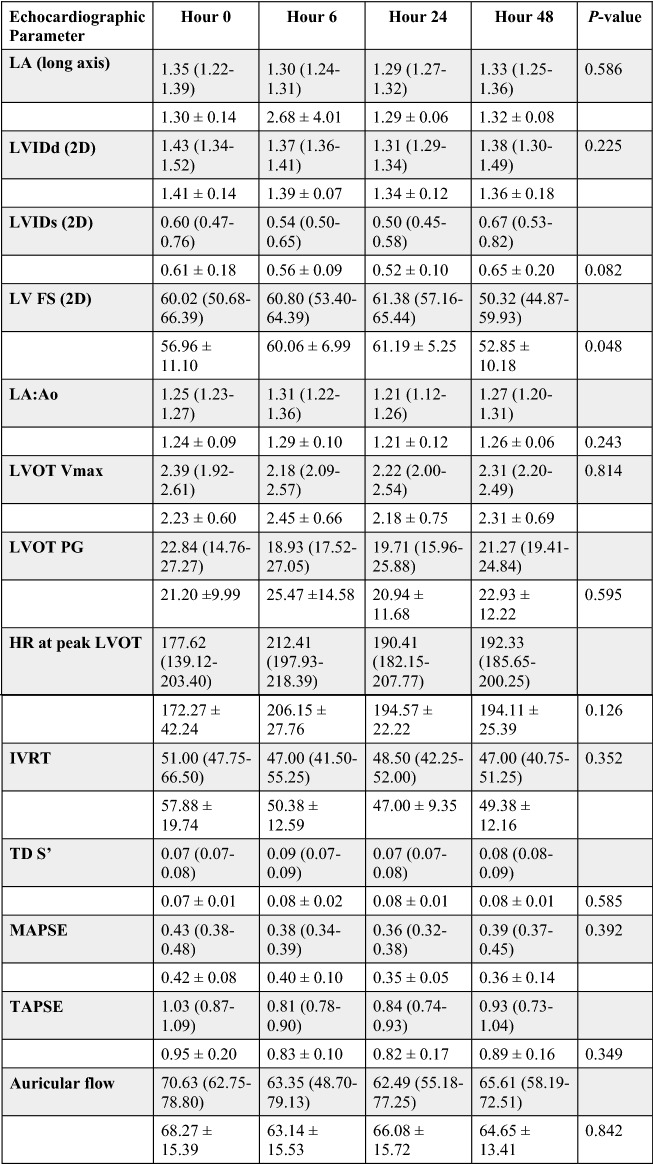
Treatment group A: Vehicle.

Echocardiographic variables for the vehicle treatment group with reported mean ± standard deviation (unshaded rows) and median and interquartile range (shaded rows). The *P*_value_ for variables that were statistically evaluated by either repeated measures one-way ANOVA or Friedman’s test are shown.

*LA* left atrium, *LVIDd* left ventricular internal dimensions (diastole), *LVIDs* left ventricular internal dimensions (systole), *LV* left ventricle, *FS* fractional shortening, *LVIDd* left ventricular internal diameter (diastole), *LVIDs* left ventricular internal diameter (systole), *LA/Ao* left atrial-to-aortic root ratio, *LVOT* left ventricular outflow tract, *Vmax* velocity (maximum), *PG* pressure gradient, *HR* heart rate, *IVRT* isovolumic relaxation time, *TD* tissue Doppler, *S’* mitral annular systolic velocity, *MAPSE* mitral annular plane systolic excursion, *TAPSE* tricuspid annular plane systolic excursion.

**Table 3:**
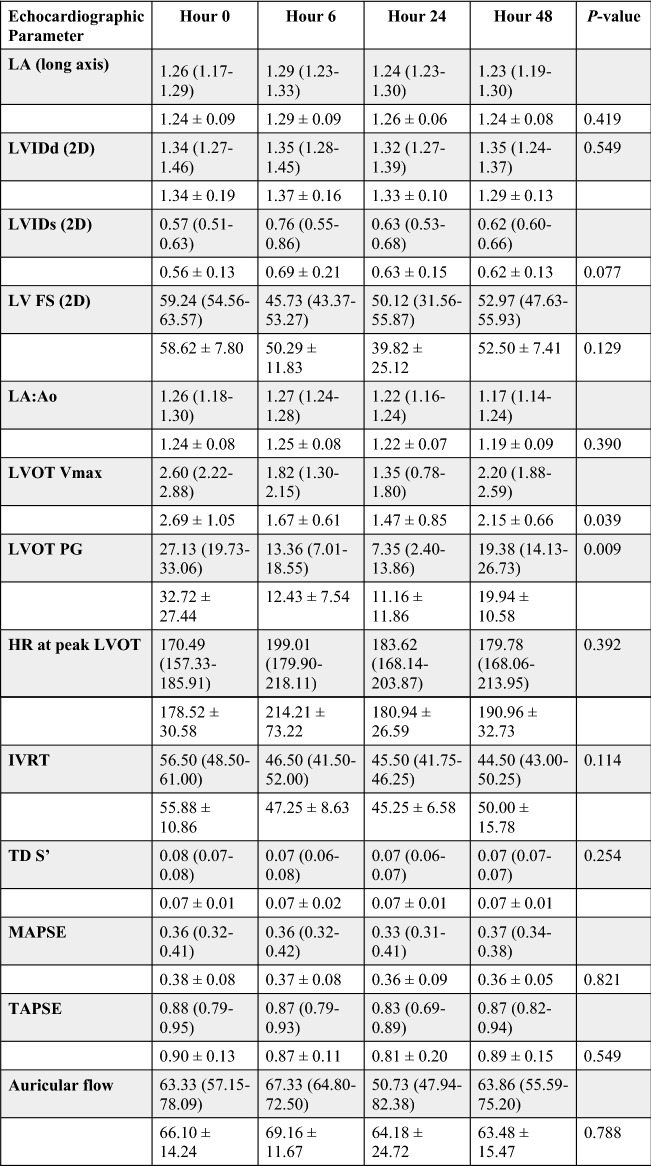
Treatment group B: 0.3 mg/kg.

Echocardiographic variables for the 0.3 mg/kg *aficamten* treatment group with reported mean ± standard deviation (unshaded rows) and median and interquartile range (shaded rows). The *P*_value_ for variables that were statistically evaluated by either repeated measures one-way ANOVA or Friedman’s test are shown.

*LA* left atrium, *LVIDd* left ventricular internal dimensions (diastole), *LVIDs* left ventricular internal dimensions (systole), *LV* left ventricle, *FS* fractional shortening, *LVIDd* left ventricular internal diameter (diastole), *LVIDs* left ventricular internal diameter (systole), *LA/Ao* left atrial-to-aortic root ratio, *LVOT* left ventricular outflow tract, *Vmax* velocity (maximum), *PG* pressure gradient, *HR* heart rate, *IVRT* isovolumic relaxation time, *TD* tissue Doppler, *S’* mitral annular systolic velocity, *MAPSE* mitral annular plane systolic excursion, *TAPSE* tricuspid annular plane systolic excursion.

**Table 4:**
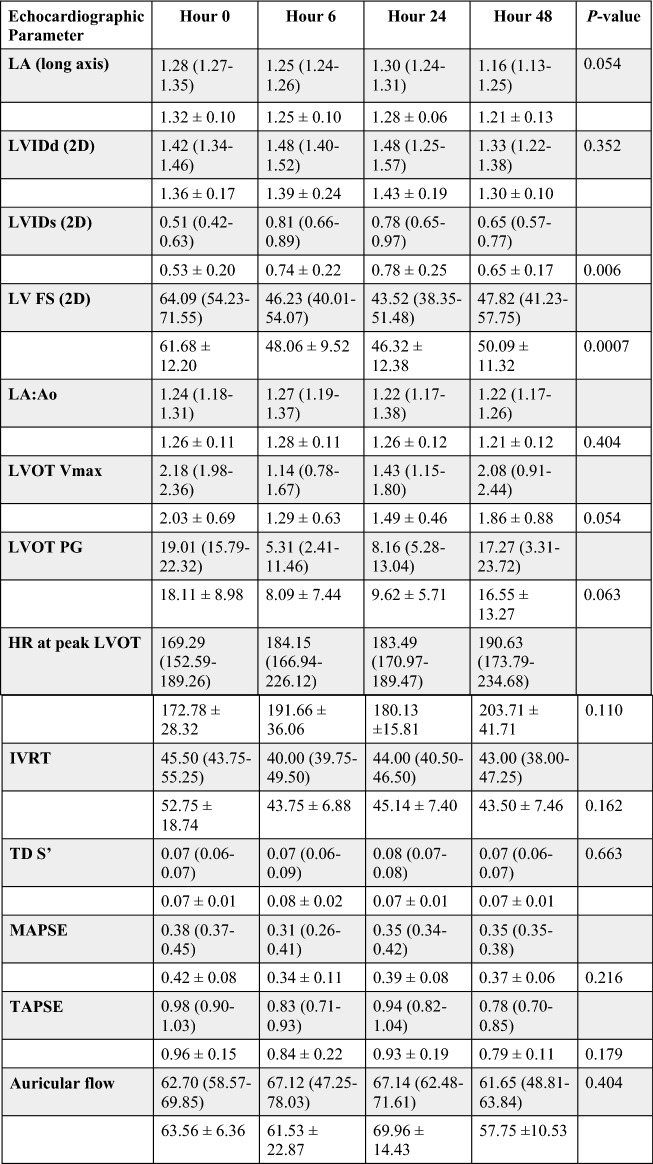
Treatment group C: 1 mg/kg.

Echocardiographic variables for the 1 mg/kg *aficamten* treatment group with reported mean ± standard deviation (unshaded rows) and median and interquartile range (shaded rows). The *P*_value_ for variables that were statistically evaluated by either repeated measures one-way ANOVA or Friedman’s test are shown.

*LA* left atrium, *LVIDd* left ventricular internal dimensions (diastole), *LVIDs* left ventricular internal dimensions (systole), *LV* left ventricle, *FS* fractional shortening, *LVIDd* left ventricular internal diameter (diastole), *LVIDs* left ventricular internal diameter (systole), *LA/Ao* left atrial-to-aortic root ratio, *LVOT* left ventricular outflow tract, *Vmax* velocity (maximum), *PG* pressure gradient, *HR* heart rate, *IVRT* isovolumic relaxation time, *TD* tissue Doppler, *S’* mitral annular systolic velocity, *MAPSE* mitral annular plane systolic excursion, *TAPSE* tricuspid annular plane systolic excursion.

There were no statistically significant differences of vehicle treated patients from baseline echocardiographic values, supporting a pharmacologic effect. No other echocardiographic parameters or heart rate (HR) was significantly different at any given time point. The plasma concentrations of *aficamten* were also monitored at the 6-, 24-, and 48-h time points and are provided in Table 5.

**Table 5:** *Aficamten* plasma concentration values at 6-, 24-, and 48-h post-dose following 0.3 and 1 mg/kg doses.

	6 h Post-dose	24 h Post-dose	48 h Post-dose
0.3 mg/kg (n = 8)	0.066 ± 0.006 µM	0.046 ± 0.005 µM	0.032 ± 0.003 µM
1 mg/kg (n = 8)	0.182 ± 0.024 µM	0.147 ± 0.016 µM	0.097 ± 0.014 µM

Plasma values are expressed as mean ± SEM.

## Discussion

A research colony of mixed-breed Maine Coon cats with the A31P *MYBPC3* mutation and a clinical diagnosis of oHCM was used to demonstrate effects of a single oral administration of two different doses of a next-in-class cardiac myosin inhibitor, *aficamten*. Treatment with *aficamten* resulted in dose-dependent reductions in LV systolic function secondary to increased LVIDs and preserved LVIDd. A previous study in this translational model demonstrated a significant decrease in LV FS% and LVOTO with a significant increase in HR six hours following administration of *aficamten* at a higher dose (2 mg/kg)^22^. This finding is thought to represent a reflex tachycardia from reduced systolic function. The current study utilized lower doses (0.3 mg/kg and 1 mg/kg) of *aficamten* and demonstrated similar beneficial effects without significant changes in HR, suggesting that the optimal therapeutic dose in oHCM cats is less than 2 mg/kg^23^.

Hypertrophic cardiomyopathy is the most prevalent inherited cardiac disease in humans with an estimated incidence of up to 1:500 (0.2%) in the general population^5^. Despite the prevalence of this disease, advancements in therapeutic options are severely lacking and treatment is based on empirical therapy with drugs that are not disease specific. For those with severe refractory symptoms, reliance on invasive techniques to relieve outflow tract obstruction and improve symptoms remains the only option^15, 24^. One limitation in the study of novel pharmaceuticals is the need for an appropriate translational model. A valuable large animal model for heart failure with preserved ejection fraction (HFpEF) has been developed via aortic banding in cats,however, this model does not precisely mimic the inappropriate molecular interactions that occur secondary to the genetic mutations that contribute to HCM^25^. Cats with spontaneously occurring HCM circumvent these limitations as they have an analogous molecular basis for disease^26^^,^^27^. Furthermore, the presence of naturally occurring LVOTO is not found in murine models of HCM, making the study of drugs aiming to influence obstruction ideal for evaluation in the feline model.

The identification and treatment of LVOTO is important in humans with oHCM as it likely contributes to symptoms and is an independent predictor of progression to severe symptoms of heart failure and death^14^. Current therapies including beta-blockers, calcium channel blockers, and disopyramide which have limited effect and substantial side effect burden human population, and have also failed to demonstrate a clinical benefit in cats with HCM^24, 28^. Additional therapies include anti-arrhythmic and anti-coagulant medications that target prevention of secondary effects of HCM (i.e., arrhythmias and thromboembolic complications) rather than the underlying myocardial disease^5, 29, 30^. In human patients who continue to experience shortness of breath, angina, or syncope, despite current medical options, invasive procedures (surgery and alcohol septal ablation) aimed as relief of obstruction can be considered but come with risk of complications, including mortality, and incomplete response^31^. However, all of the aforementioned therapies are focused on relieving symptoms rather than targeting the underlying pathophysiologic mechanisms of HCM.

The development of novel cardiac myosin inhibitors is a unique and promising new frontier for targeted therapies in affected individuals and may improve clinical signs as well as prevent deleterious effects of abnormal actin and myosin interactions, leading to reduced histopathologic changes and delayed progression of disease. A study in mice with HCM demonstrated the ability of another small molecule inhibitor, mavacamten, to significantly reduce the hallmark histopathologic features of fibrosis and myocardial disarray when administered prior to the development of significant hypertrophy^21^. Additionally, a previous study of mavacamten in this feline research colony demonstrated reduction of LVOTO using a provokable model of isoproterenol induced LVOTO under anesthesia^20^. Our results demonstrate that *aficamten* also significantly reduces LV systolic function and relieves LVOTO in the same population. Further studies with an induced LVOTO model can be considered and may result in even more marked improvement in obstruction. Studies of long-term administration with potential for eventual histopathologic evaluation are also warranted to further investigate the microstructural effects of therapy on prevention of detrimental remodeling.

Safety and tolerability of *aficamten* was demonstrated in a Phase I study in healthy adults, allowing initiation of a Phase II study (REDWOOD-HCM) that demonstrated significant reductions in peak resting and provoked (post-Valsalva) LV outflow pressure gradients with only modest reductions in LV ejection fraction^2^. Mavacamten was evaluated in a phase III clinical trial, EXPLORER-HCM, that demonstrated treatment was well tolerated and improved LVOTO, exercise capacity, and NYHA functional classification of heart failure. A second phase III clinical trial (VALOR-HCM) demonstrated short term efficacy of mavacamten in reducing the proportion of patients meeting indications for invasive septal reduction therapy in oHCM patients previously indicated for the procedures^32^^,^^33^. Our study demonstrates the safety and tolerability of a single oral administration of *aficamten* at doses 0.3 mg/kg and 1.0 mg/kg in cats. These findings highlight the need for continued investigation of small molecule inhibitors while demonstrating a unique opportunity for valuable therapeutic potential in the preclinical and clinical stages that may improve clinical signs and prevent disease progression.

Our study also demonstrates the plasma concentrations of *aficamten* at each time point following each dose administration in the feline model. The most profound effects of LV systolic function were noted at six hours, when the highest plasma concentrations were noted. The goals for development of this second-generation myosin inhibitor are to provide a predictable half-life in humans for once daily dosing that reaches a steady state within two weeks, to maintain a wide therapeutic index and to avoid substantial effects on cytochrome P450. *Aficamten* has demonstrated these beneficial traits and long term dosing with further pharmacokinetic/pharmacodynamic studies may be beneficial in cats^34^.

Several limitations to this study exist. There is the possibility of type II error given the small sample size of eight cats. The temperament of the colony cats and drug administration by oral gavage requires significant sedation which may impact functional parameters. To minimize these effects, a three-hour recovery period was allowed following sedation with alfaxalone for oral gavage and a standardized sedation protocol (butorphanol and acepromazine) that previously demonstrated minimal effects on echocardiographic variables was used for each echocardiographic evaluation^35^^,^^36^. Additionally, the effect of repeated evaluations may mean that acclimation to the environment could have an effect on sympathetic tone and stress. This is perhaps an explanation for the reduced left ventricular fractional shortening seen in the vehicle group overall, however this change was not found in association with alteration of heart rate, and was found to be insignificant between all groups when Tukey’s multiple comparison testing was applied.

The development of novel small molecule inhibitors is an exciting frontier for the advancement of therapeutic options in the management of preclinical HCM; the current study further highlights the utility of this feline model in evaluation of novel therapies that may benefit the human population. Based on the current study, the optimal dose of *aficamten* appears to be in the 0.3–1 mg/kg range in cats. A single dose of *aficamten* significantly decreased LV systolic function and improved LVOTO, both of which are harmful aspects of this disease process that contribute to the pathogenesis. Future studies are warranted to evaluate the effects of long-term administration of *aficamten* on the prevention of disease progression in the asymptomatic period of disease. Studies investigating the effects of chronic target dosing are warranted and this translational model of HCM may provide valuable continued insight to the investigation of this next-in-class therapy in the human disease.

## Methods

### Animals

All experimental protocols were approved by the University of California Davis Institutional Animal Care and Use Committee (IACUC). Research was conducted in accordance with all relevant guidelines and regulations of the University of California, Davis, IACUC (Protocol number 20565) and all methods are reported in accordance with ARRIVE guidelines. A sample size of at least 7 cats in each dose group was calculated to have an 80% power to identify a 15% reduction in key parameters of echocardiographically-assessed systolic function. This a-priori sample size was estimated based upon colony-specific, preliminary data in HCM-affected feline subjects^37^. Thus, eight purpose-bred mixed-breed Maine Coon cats with a naturally occurring A31P *MYBPC3* mutation coupled with a diagnosis of subclinical oHCM were used in this study. The cats were group housed in the feline research colony at the University of California, Davis (Davis, CA, USA) throughout the study period and were maintained on the same dietary regime without supplementation or medication beyond the study protocol. Cats were transported to the laboratory facilities and individually housed for the time of the study procedures and until full recovery from any sedation procedure was observed prior to return to the colony. Physical examinations were performed on all animals and no clinical signs of their known HCM were appreciated.

### Experimental design

This was a randomized, controlled, cross-over study. Cats received single oral doses of vehicle or *aficamten* at a dose of 0.3 mg/kg or 1.0 mg/kg via oral gavage in 0.5% (w/v) hydroxypropyl methylcellulose (HPMC) and 0.1% TWEEN® 80 in UltraPure water at a dose volume of 1 mg/kg. Subsequent echocardiographic evaluations were performed at baseline, 6, 24 and 48 h after administration. Cats were randomized and crossed over to the other treatment group so that all cats received vehicle and both treatments with a minimum washout period of 2 weeks as detailed in Table 6.

**Table 6:** Cross-over study design.

Subject and dose				
Week 1	Cat 1–1 mg/kg	Cat 2 – 0.3 mg/kg	Cat 3–1 mg/kg	Cat 4 – 0.3 mg/kg
Week 2	Cat 5–Vehicle	Cat 6 – Vehicle	Cat 7 – 1 mg/kg	Cat 8 Vehicle
Week 3	Cat 1 – 0.3 mg/kg	Cat 2 – Vehicle	Cat 3 – Vehicle	Cat 4 – 1 mg/kg
Week 4	Cat 5 – 0.3 mg/kg	Cat 6 – 1 mg/kg	Cat 7 – 0.3 mg/kg	Cat 8 – 1 mg/kg
Week 5	Cat 1 – Vehicle	Cat 2 – 1 mg/kg	Cat 3 – 0.3 mg/kg	Cat 4—Vehicle
Week 6	Cat 5 – 1 mg/kg	Cat 6 – 0.3 mg/kg	Cat 7 – Vehicle	Cat 8 – 0.3 mg/kg

### Echocardiographic evaluations

Baseline echocardiographic evaluations were performed 24 h prior to each treatment dosing. On the morning of the examination, cats were dosed with gabapentin (100 mg PO) approximately one hour prior to the study. The cats were sedated with butorphanol (0.3 mg/kg IM) and acepromazine (0.1–0.5 mg/kg IM) to facilitate echocardiographic evaluation.

All echocardiograms were performed by a single board-certified cardiologist (MSO) using a 12–4 mHz sector array transducer with harmonics (Philips EPIC CVx, Philips Healthcare, Andover, MA, USA). Two-dimensional (2D), M-mode, color Doppler, and spectral Doppler echocardiographic images were obtained in standard imaging planes from right and left lateral recumbency^38^. A concurrent lead II ECG was monitored for arrhythmias.

On the day of echocardiographic evaluation with vehicle or *aficamten* treatment, cats were sedated with alfaxalone (2 mg/kg IM) to facilitate oral gavage of one dose of specified treatment (vehicle, 0.3 mg/kg, or 1 mg/kg *aficamten*) followed by a three-hour recovery period. To facilitate echocardiographic evaluation, cats were sedated with butorphanol (0.3 mg/kg IM) and acepromazine (0.1–0.5 mg/kg IM) at the 6-, 24-, and 48-h post-dose echocardiography time points. All appropriate cardiac views were obtained for 2-D, M-Mode, color, and spectral Doppler assessment of LV hypertrophy, LVOTO, LV fractional shortening (FS%), indices of diastolic function, and other parameters. Following echocardiographic evaluation, blood samples were obtained for plasma concentration analysis. Cats were observed and allowed to fully recover before returning to colony group housing.

Images were stored and all measurements were performed by a single blinded observer (MSO) using a commercially available offline workstation (Syngo Dynamic Workplace v10.0.01_HF04_Rev5 [Build 2884], Siemens Medical Solutions, Malvern, PA, USA). Two-dimensional right parasternal long axis or short axis imaging planes were measured to obtain the maximum 2D thickness of the interventricular septum (IVSd) and left ventricular posterior wall (LVPWd) in diastole using an inner-edge to inner-edge measuring technique. Segmental or diffuse IVS or LVPW thickness exceeding 6 mm in the absence of systemic hypertension or hyperthyroidism on at least two serial examinations > 1 month apart was considered consistent with HCM^19^. Left ventricular outflow tract obstruction was identified from the left parasternal 5-chamber view and was defined as the presence of color Doppler flow aliasing in the LVOT and a late-peaking spectral CW Doppler signal with a velocity of > 1.9 m/s^38^^,^^39^. The cursor was aligned with the color Doppler aliasing flow and the maximal modal velocity obtained was recorded.

M-mode and two-dimensional right parasternal short axis imaging planes were used to measure the left ventricular internal dimensions at end-diastole (LVIDd) and end-systole (LVIDs). Fractional shortening was calculated using the equation (LVIDd-LVIDs)/LVIDd × 100.

Left atrial (LA) size was measured in 2D on the right parasternal short axis basilar view to determine the LA/Ao as previously described^40^. The maximal LA diameter in long axis was measured from the right parasternal long axis view^41^. The left auricular flow velocity was obtained in an oblique left apical parasternal long axis view with the pulsed wave Doppler sample volume positioned at the entrance to the left auricle^42^.

Spectral Doppler and pulsed wave tissue Doppler imaging (PW TDI) from the left apical 4-chamber view were used to evaluate diastolic functional parameters including transmitral inflow patterns, lateral E’ and A’, and LV isovolumic relaxation time (IVRT)^40^. Lateral mitral annulus S’ was measured from this view with PW TDI and mitral annular plane systolic excursion (MAPSE) was measured with M-mode. Transmitral spectral Doppler and diastolic tissue Doppler was frequently fused with tachycardia and precludes measurements of these values.

### Blood sample collection and plasma analysis

Following each echocardiographic assessment (baseline, 6-, 24-, and 48-h post-dose), 2 mL of whole blood was collected via direct venipuncture. Blood samples were collected into EDTA microtainer tubes and centrifuged for 5 min at 5000 rpm to obtain the plasma component. Plasma was stored frozen at − 80 °C until subsequent *aficamten* concentration measurements.

*Aficamten* plasma concentration was determined by liquid chromatography with tandem mass spectrometry (LC–MS/MS) as previously described^43^. Briefly, a 25 µL aliquot of each plasma sample was diluted with 25 µL blank cat plasma. The plasma sample was then mixed with 100 µL of acetonitrile that contained N1-(butylcarbamoyl)-sulfanamide (0.3 µM) as the internal standard. The mixture was then vortexed and centrifuged. The resulting supernatant was transferred and filtered through a Whatman 96-well 0.45 µm hydrophilic PVDF unifilter membrane plate (Cytiva, Marlborough, MA, USA). 10 µL of the resulting solution was injected onto a reverse-phase C18 column and the resultant peaks detected on a SCIEX API 4000 LC–MS/MS equipped with a turbo ion spray ionization source.

### Statistics

Data was tested for normality by visual inspection and the D’Agostino and Pearson Omnibus normality test. Descriptive statistics were performed to provide both the mean and standard deviation for parametric data, and the median and interquartile range for non-parametric data. Data was compared between vehicle and *aficamten* treatment (0.3 mg/kg and 1 mg/kg) across each of the four time points (0-, 6-, 24-, and 48-h post-dose). When all data sets being compared were normally distributed, a repeated measures one-way ANOVA test was performed to identify differences between time points. When the repeated measures one-way ANOVA resulted in a *P* < 0.05, the post-hoc Tukey’s multiple comparisons test was performed to identify which, if any, time points were statistically different from each other. When all data sets being compared were not normally distributed, a Friedman test was performed to identify differences between time points. When the Friedman test resulted in a *P* < 0.05, the post-hoc Dunn’s multiple comparisons test was performed to identify which, if any, time points were statistically different from each other. For all statistical testing, *P* < 0.05 was considered statistically significant.

## Data Availability

Restrictions apply to the availability of these data. Data are available from the corresponding author with the permission of Cytokinetics, Inc.
